# The zwitterion (23′*E*)-(23*R*,25*S*)-23-[1-(oxidoiminio)eth­yl]-5β-spiro­stan-3β-yl acetate

**DOI:** 10.1107/S1600536809044651

**Published:** 2009-10-31

**Authors:** María-Guadalupe Hernández Linares, Jesús Sandoval Ramírez, Socorro Meza Reyes, Sara Montiel Smith, Sylvain Bernès

**Affiliations:** a Escuela de Ingeniería Química, Universidad del Istmo, Ciudad Universitaria s/n, 70760 Sto. Domingo Tehuantepec, Oax., Mexico; b Facultad de Ciencias Químicas, Benemérita Universidad Autónoma de Puebla, Ciudad Universitaria, San Manuel, 72000 Puebla, Pue., Mexico; c DEP Facultad de Ciencias Químicas, UANL, Guerrero y Progreso S/N, Col. Treviño, 64570 Monterrey, N.L., Mexico

## Abstract

The title steroidal compound, C_31_H_49_NO_5_, resulted from the selective oximation of (23*R*)-23-acetyl­sarsasapogenin acetate. One- and two-dimensional ^1^H and ^13^C NMR spectra, as well as IR data, are in agreement with the presence of a ketoxime group at C-23. However, recrystallization in slightly acidic media affords the title compound in the rare zwitterionic oxime form, as a consequence of migration of the hydr­oxy H atom to the N atom in the oxime group. This H atom is clearly detected and its position was refined from X-ray data. The geometry for the C=N^+^(H)—O^−^ group features long C=N and short N—O bond lengths compared to non-zwitterionic oximes. The ketoxime is stabilized with the *E* configuration, avoiding steric hindrance between the oxime O atom and H atom at C-23. The sum of the angles around the oxime N atom is 359.6°, giving a planar configuration for that atom, as expected for *sp* ^2^ hybridization.

## Related literature

For the synthesis of (23*R*)-23-acetyl­sarsasapogenin acetate used as starting material, see: Meza-Reyes *et al.* (2005 ▶). For the full spectroscopic characterization of the tautomers of the title compound, see: Hernández-Linares (2005 ▶). For tautomerism between oximes and imine *N*-oxides, see: Fernández *et al.* (1994 ▶). For related zwitterionic oximes and hydro­chloride oximes characterized by X-ray diffraction, see: Witte *et al.* (1984 ▶); Fernández *et al.* (1994 ▶); Gurkova *et al.* (1988 ▶); Laus *et al.* (2008 ▶); Forgan *et al.* (2008 ▶).
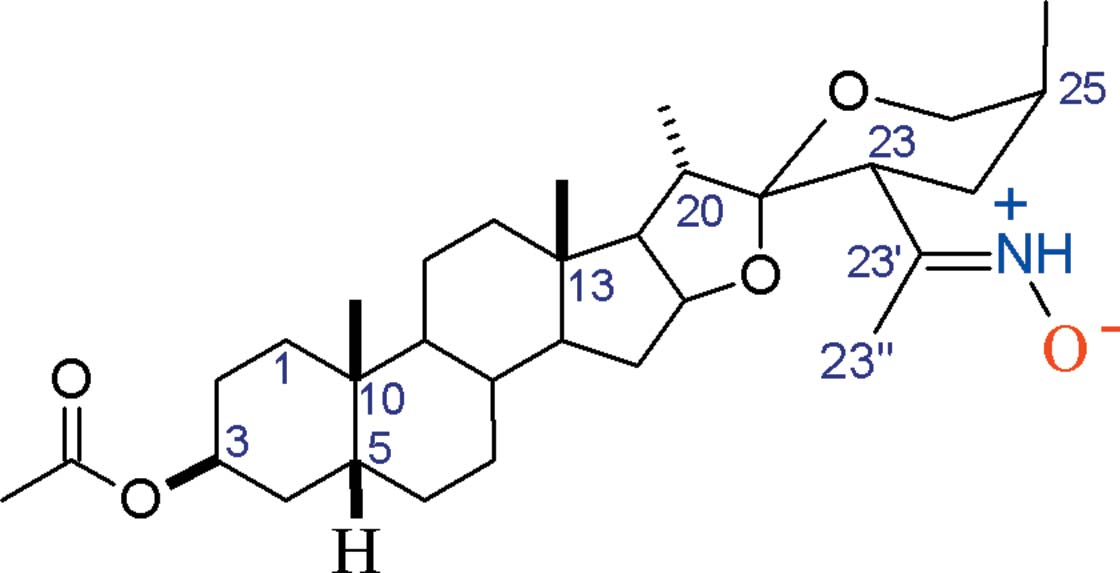


## Experimental

### 

#### Crystal data

C_31_H_49_NO_5_
*M* *_r_* = 515.71
Orthorhombic, 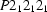

*a* = 8.385 (2) Å
*b* = 11.5627 (16) Å
*c* = 30.420 (5) Å
*V* = 2949.2 (10) Å^3^
*Z* = 4
Mo *K*α radiation
μ = 0.08 mm^−1^
*T* = 298 K
0.60 × 0.60 × 0.35 mm

#### Data collection

Bruker P4 diffractometer
Absorption correction: none
4182 measured reflections
2956 independent reflections
2512 reflections with *I* > 2σ(*I*)
*R*_int_ = 0.041
3 standard reflections every 97 reflections intensity decay: 3%

#### Refinement

*R*[*F* ^2^ > 2σ(*F* ^2^)] = 0.048
*wR*(*F* ^2^) = 0.129
*S* = 1.02
2956 reflections
344 parameters
2 restraints
H atoms treated by a mixture of independent and constrained refinement
Δρ_max_ = 0.46 e Å^−3^
Δρ_min_ = −0.25 e Å^−3^

### 

Data collection: *XSCANS* (Siemens, 1994 ▶); cell refinement: *XSCANS*; data reduction: *XSCANS*; program(s) used to solve structure: *SHELXTL* (Sheldrick, 2008 ▶); program(s) used to refine structure: *SHELXTL*; molecular graphics: *SHELXTL*; software used to prepare material for publication: *SHELXTL*.

## Supplementary Material

Crystal structure: contains datablocks I, global. DOI: 10.1107/S1600536809044651/nk2010sup1.cif

Structure factors: contains datablocks I. DOI: 10.1107/S1600536809044651/nk2010Isup2.hkl

Additional supplementary materials:  crystallographic information; 3D view; checkCIF report

## Figures and Tables

**Table:** 

C32—C33	1.477 (5)
C32—N34	1.361 (6)
N34—H34	0.980 (18)
N34—O35	1.367 (5)

**Table:** 

C23—C32—C33	122.0 (3)
N34—C32—C23	111.6 (3)
N34—C32—C33	126.3 (3)
C32—N34—O35	109.0 (4)
C32—N34—H34	132.0 (16)
O35—N34—H34	118.6 (16)

**Fig. 1.**
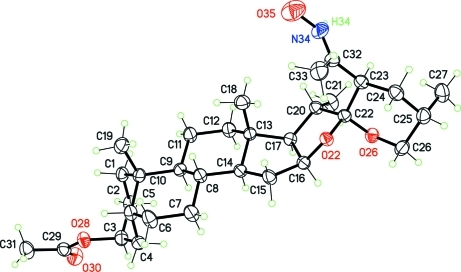
The title molecule with displacement ellipsoids for non-H atoms shown at the 30% probability level.

## supplementary crystallographic information

### Comment

Synthetic routes are available for the large scale preparation of (23*R*)-23-acetylsarsasapogenin acetate (Meza-Reyes *et al.*, 2005). During attempts to functionalize the 23 position in sarsasapogenin acetate, we have found that the oximation of (23*R*)-23-acetylsarsasapogenin acetate using NH_2_OH.HCl afforded the expected 23-hydroxyimino derivative. IR spectrum for that compound exhibits a strong vibration at 3400 cm^-1^ (*ν*_O—H_), a weak vibration at 1636 cm^-1^ (*ν*_C=N_), and a vibration at 951 cm^-1^ (*ν*_N—O_), characteristics of ketoximes. One-dimensional and two-dimensional ^1^H and ^13^C NMR data are also in full agreement with that assignment (Hernández-Linares, 2005, and archived CIF). For instance, 31 signals are detected in ^13^C-NMR, one of which, at 159.3 p.p.m., is characteristic of the C23^1^ atom substituted by an hydroxyimino group.

However, recrystallization of the ketoxime in a slightly acidic medium (MeOH/CH_2_Cl_2_, 98:2) afforded a solid with a significantly different melting point (459 K for the oxime, 516 K for the recrystallized material). On the other hand, the high m.p. solid presents a new ^1^H-NMR broad signal at 9.05 p.p.m., and the stretching vibration for OH group in the IR spectrum no longer appears, while a new strong vibration is observed at 2300 cm^-1^. In order to rationalize these dramatic changes, the X-ray analysis of this material was carried out.

The molecular structure (Fig. 1) resolves the above mentioned problem: a difference map shows a strong residual in the vicinity of atom N34, while no H atom is found bonded to O35. The oxime is thus stabilized in the solid-state in a rather rare zwittterionic form. The residual close to N34 refines well as an H atom. The N—H bond accounts for the IR vibration at 2300 cm^-1^ and H34 is detected in ^1^H-NMR at 9.05 p.p.m..

Refinement of the proposed model was however not so straightforward. Alternative models assuming a non-zwitterionic oxime were probed, with disordered N and O sites, which resulted in oxime groups with geometry far from expectation and physically unreasonable intermolecular contacts. On the other hand, refinements carried out with a free H34 atom converged to a geometry where the oxime N atom deviates significantly from the expected planar trigonal arrangement, although the N34—H34 bond length lies in the expected range. Considering that the structure is based on room-temperature data, we eventually refined the structural model with a pair of soft restraints for non-bonding separations involving H34 (see *Experimental*). Finally, a supplementary concern is about the terminal O atom O35, which is separated by 2.757 (5) Å from the carbonyl O30 atom of a symmetry-related molecule (symmetry code: 2 - *x*, -1/2 + *y*, 1/2 - *z*). Such an arrangement would be suitable for a stabilizing hydrogen bond, which is not observed because of the migration of the oxime H atom to N34. With the currently available data, it is however difficult to decide if this situation resulted from the actual migration of the H atom in the solid-sate. Definitively, more data are required in order to fully characterize this zwitterion, for example low temperature neutron diffraction data, and the X-ray structure of the non-zwitterionic title molecule.

The tautomerism between oximes and imine *N*-oxides seems to be poorly documented (Fernández *et al.*, 1994). In the case of the title compound, the C═N bond length is long, 1.361 (6) Å, and the N—O bond length is short, 1.367 (5) Å, compared to common non-zwitterionic oximes (*ca*. 1.28 and 1.41 Å, respectively). The sum of angles around N2 is very close to 360°, as expected for a *sp*^2^ hybridized atom. Therefore, delocalization is extended on the whole hydroxyimino group, C32/N34/O35, which is almost planar. The imine group presents the *E* configuration, which avoids steric hindrance between the oxime O atom and methine H atom H23C. This geometry is close to that previously reported for the few zwitterionic oximes or hydrochloride oximes characterized by X-ray diffraction (Fernández *et al.*, 1994; Gurkova *et al.*, 1988; Laus *et al.*, 2008). The imine *N*-oxide tautomer has also been used as a ligand (Witte *et al.*, 1984; Forgan *et al.*, 2008).

### Experimental

In a 100 ml round bottom flask was dissolved 2 mmol (1 g) of (23*R*)-23-acetylsarsasapogenin acetate in ethanol (30 ml). Pyridine (1 ml) and 2 mmol of NH_2_OH.HCl (0.139 g) were added. The mixture was refluxed for 3 h, following the reaction by TLC. Solvent was then eliminated under reduced pressure, affording the crude oxime. The crude was recrystallized in a mixture of MeOH/CH_2_Cl_2_ (98:2), yielding the iminium olate zwitterion (96%). When the crude product was neutralized and then crystallized, the hydroxyimino derivative was obtained Anal. found (calc. for C_31_H_49_NO_5_): C 72.20 (72.20), H 9.56 (9.57), N 2.71 (2.71%).

### Refinement

H34 was found in a difference map and refined with free coordinates, although geometry around N34 was restrained through soft restraints in order to avoid significant deviations from trigonal geometry. The X-ray structure of acetone oxime hydrochloride was used as target (Gurkova *et al.*, 1988): H34···C32 and H34···O35 separations were restrained to 2.10 (2) Å. The isotropic displacement parameter for H34 was fixed to *U*_iso_(H34) = 1.5*U*_eq_(N34). Other H atoms were placed in idealized positions and refined using a riding model, with fixed C—H bond lengths: 0.96 (methyl), 0.97 (methylene) or 0.98 Å (methine). Isotropic displacement parameters were computed as *U*_iso_(H) = 1.5*U*_eq_(carrier C) for methyl groups and *U*_iso_(H) = 1.2*U*_eq_(carrier C) for other H atoms. Methyl groups were allowed to rotate about their C—C bonds.

### Crystal data

**Table:**

C_31_H_49_NO_5_	*D*_x_ = 1.161 Mg m^−^^3^
*M**_r_* = 515.71	Melting point: 516 K
Orthorhombic, *P*2_1_2_1_2_1_	Mo *K*α radiation, λ = 0.71073 Å
Hall symbol: P 2ac 2ab	Cell parameters from 44 reflections
*a* = 8.385 (2) Å	θ = 4.7–12.5°
*b* = 11.5627 (16) Å	µ = 0.08 mm^−^^1^
*c* = 30.420 (5) Å	*T* = 298 K
*V* = 2949.2 (10) Å^3^	Irregular, colourless
*Z* = 4	0.60 × 0.60 × 0.35 mm
*F*(000) = 1128	

### Data collection

**Table:**

Bruker P4 diffractometer	*R*_int_ = 0.041
Radiation source: fine-focus sealed tube	θ_max_ = 25.0°, θ_min_ = 1.9°
graphite	*h* = −9→2
ω scans	*k* = −13→1
4182 measured reflections	*l* = −36→1
2956 independent reflections	3 standard reflections every 97 reflections
2512 reflections with *I* > 2σ(*I*)	intensity decay: 3%

### Refinement

**Table:**

Refinement on *F*^2^	Secondary atom site location: difference Fourier map
Least-squares matrix: full	Hydrogen site location: inferred from neighbouring sites
*R*[*F*^2^ > 2σ(*F*^2^)] = 0.048	H atoms treated by a mixture of independent and constrained refinement
*wR*(*F*^2^) = 0.129	*w* = 1/[σ^2^(*F*_o_^2^) + (0.0651*P*)^2^ + 1.0057*P*] where *P* = (*F*_o_^2^ + 2*F*_c_^2^)/3
*S* = 1.02	(Δ/σ)_max_ < 0.001
2956 reflections	Δρ_max_ = 0.46 e Å^−^^3^
344 parameters	Δρ_min_ = −0.25 e Å^−^^3^
2 restraints	Extinction correction: *SHELXTL* (Sheldrick, 2008), Fc^*^=kFc[1+0.001xFc^2^λ^3^/sin(2θ)]^-1/4^
Primary atom site location: structure-invariant direct methods	Extinction coefficient: 0.0095 (18)

### Fractional atomic coordinates and isotropic or equivalent isotropic displacement parameters (Å^2^)

**Table:**

	*x*	*y*	*z*	*U*_iso_*/*U*_eq_	
C1	1.0856 (4)	0.6448 (3)	0.33730 (11)	0.0516 (9)	
H1A	1.0915	0.5917	0.3619	0.062*	
H1B	1.1393	0.6086	0.3126	0.062*	
C2	1.1766 (4)	0.7539 (4)	0.34940 (12)	0.0560 (9)	
H2D	1.1862	0.8030	0.3237	0.067*	
H2E	1.2832	0.7333	0.3589	0.067*	
C3	1.0929 (5)	0.8205 (3)	0.38583 (11)	0.0554 (9)	
H3A	1.1486	0.8934	0.3917	0.066*	
C4	0.9219 (5)	0.8431 (3)	0.37427 (11)	0.0531 (9)	
H4A	0.9182	0.8964	0.3497	0.064*	
H4B	0.8701	0.8802	0.3991	0.064*	
C5	0.8285 (4)	0.7338 (3)	0.36217 (10)	0.0485 (8)	
H5A	0.8271	0.6848	0.3885	0.058*	
C6	0.6548 (4)	0.7627 (4)	0.35160 (11)	0.0599 (11)	
H6A	0.6116	0.8113	0.3747	0.072*	
H6B	0.5929	0.6919	0.3508	0.072*	
C7	0.6396 (5)	0.8252 (4)	0.30744 (11)	0.0587 (10)	
H7B	0.5277	0.8390	0.3012	0.070*	
H7C	0.6925	0.8996	0.3093	0.070*	
C8	0.7128 (4)	0.7551 (3)	0.27006 (10)	0.0447 (8)	
H8B	0.6522	0.6831	0.2670	0.054*	
C9	0.8885 (4)	0.7238 (3)	0.27973 (10)	0.0421 (7)	
H9B	0.9461	0.7972	0.2821	0.051*	
C10	0.9083 (4)	0.6615 (3)	0.32507 (10)	0.0441 (8)	
C11	0.9635 (5)	0.6585 (3)	0.24098 (10)	0.0552 (9)	
H11B	0.9145	0.5827	0.2389	0.066*	
H11C	1.0761	0.6473	0.2469	0.066*	
C12	0.9452 (4)	0.7207 (3)	0.19660 (11)	0.0519 (9)	
H12A	1.0078	0.7913	0.1970	0.062*	
H12B	0.9866	0.6716	0.1734	0.062*	
C13	0.7726 (4)	0.7502 (3)	0.18680 (10)	0.0410 (7)	
C14	0.7059 (4)	0.8196 (3)	0.22627 (10)	0.0434 (8)	
H14B	0.7764	0.8867	0.2296	0.052*	
C15	0.5478 (4)	0.8673 (4)	0.20928 (10)	0.0531 (9)	
H15B	0.5123	0.9329	0.2266	0.064*	
H15C	0.4655	0.8084	0.2093	0.064*	
C16	0.5917 (4)	0.9040 (3)	0.16224 (10)	0.0448 (8)	
H16B	0.6064	0.9880	0.1607	0.054*	
C17	0.7482 (4)	0.8411 (3)	0.14966 (10)	0.0405 (7)	
H17C	0.8361	0.8968	0.1508	0.049*	
C18	0.6769 (5)	0.6401 (3)	0.17818 (12)	0.0576 (9)	
H18C	0.6759	0.5932	0.2042	0.086*	
H18D	0.7251	0.5976	0.1545	0.086*	
H18E	0.5695	0.6602	0.1704	0.086*	
C19	0.8329 (5)	0.5408 (3)	0.32368 (13)	0.0615 (10)	
H19A	0.8376	0.5066	0.3524	0.092*	
H19B	0.8903	0.4932	0.3032	0.092*	
H19C	0.7237	0.5471	0.3145	0.092*	
C20	0.7218 (4)	0.8046 (3)	0.10144 (10)	0.0435 (8)	
H20A	0.7122	0.7202	0.1007	0.052*	
C21	0.8573 (4)	0.8390 (4)	0.07093 (12)	0.0606 (10)	
H21B	0.8276	0.8230	0.0411	0.091*	
H21C	0.9512	0.7956	0.0783	0.091*	
H21D	0.8787	0.9201	0.0741	0.091*	
C22	0.5588 (4)	0.8561 (3)	0.08961 (10)	0.0391 (7)	
O22	0.4759 (2)	0.86704 (19)	0.13038 (6)	0.0415 (5)	
C23	0.4554 (4)	0.7843 (3)	0.05791 (10)	0.0430 (8)	
H23C	0.5201	0.7697	0.0317	0.052*	
C24	0.3086 (4)	0.8530 (3)	0.04293 (11)	0.0511 (8)	
H24C	0.2537	0.8100	0.0201	0.061*	
H24D	0.2359	0.8616	0.0675	0.061*	
C25	0.3526 (5)	0.9721 (3)	0.02546 (11)	0.0541 (9)	
H25C	0.2538	1.0157	0.0209	0.065*	
C26	0.4476 (5)	1.0321 (3)	0.06089 (12)	0.0552 (9)	
H26D	0.4773	1.1088	0.0509	0.066*	
H26E	0.3821	1.0405	0.0870	0.066*	
O26	0.5890 (3)	0.96787 (19)	0.07171 (7)	0.0473 (6)	
C27	0.4413 (6)	0.9665 (4)	−0.01843 (12)	0.0700 (11)	
H27C	0.4699	1.0432	−0.0275	0.105*	
H27D	0.3735	0.9319	−0.0402	0.105*	
H27E	0.5360	0.9207	−0.0151	0.105*	
O28	1.0885 (3)	0.7483 (2)	0.42577 (7)	0.0540 (6)	
C29	1.2125 (5)	0.7503 (3)	0.45297 (11)	0.0539 (9)	
O30	1.3306 (4)	0.8083 (3)	0.44684 (10)	0.0741 (8)	
C31	1.1864 (5)	0.6731 (4)	0.49129 (12)	0.0710 (12)	
H31B	1.2539	0.6970	0.5151	0.106*	
H31C	1.2117	0.5950	0.4833	0.106*	
H31D	1.0769	0.6775	0.5003	0.106*	
C32	0.4111 (4)	0.6680 (3)	0.07679 (11)	0.0483 (8)	
C33	0.2777 (5)	0.6538 (4)	0.10802 (13)	0.0660 (11)	
H33A	0.3050	0.5962	0.1295	0.099*	
H33B	0.1839	0.6299	0.0924	0.099*	
H33C	0.2574	0.7260	0.1225	0.099*	
N34	0.5075 (6)	0.5826 (3)	0.06127 (13)	0.0854 (13)	
H34	0.605 (5)	0.585 (2)	0.0434 (17)	0.128*	
O35	0.4610 (5)	0.4795 (3)	0.07914 (14)	0.1043 (12)	

### Atomic displacement parameters (Å^2^)

**Table:**

	*U*^11^	*U*^22^	*U*^33^	*U*^12^	*U*^13^	*U*^23^
C1	0.051 (2)	0.058 (2)	0.0460 (17)	0.0135 (19)	0.0002 (16)	0.0069 (16)
C2	0.0428 (18)	0.069 (2)	0.056 (2)	−0.003 (2)	−0.0010 (16)	0.013 (2)
C3	0.060 (2)	0.054 (2)	0.0515 (18)	−0.009 (2)	−0.0074 (18)	0.0068 (16)
C4	0.060 (2)	0.0538 (19)	0.0458 (17)	0.0118 (19)	−0.0014 (17)	−0.0013 (16)
C5	0.0440 (18)	0.059 (2)	0.0422 (17)	0.0067 (18)	0.0023 (15)	0.0068 (16)
C6	0.0442 (18)	0.094 (3)	0.0415 (17)	0.019 (2)	0.0072 (16)	0.003 (2)
C7	0.048 (2)	0.083 (3)	0.0454 (17)	0.027 (2)	0.0009 (16)	−0.0018 (18)
C8	0.0405 (17)	0.0530 (18)	0.0405 (16)	0.0062 (18)	0.0007 (14)	0.0011 (15)
C9	0.0392 (16)	0.0439 (17)	0.0433 (16)	0.0060 (15)	−0.0002 (14)	0.0001 (14)
C10	0.0425 (17)	0.0450 (17)	0.0449 (16)	0.0041 (16)	−0.0018 (15)	0.0045 (14)
C11	0.052 (2)	0.066 (2)	0.0474 (18)	0.019 (2)	0.0044 (16)	0.0054 (17)
C12	0.0462 (19)	0.064 (2)	0.0458 (17)	0.0113 (18)	0.0065 (16)	0.0026 (16)
C13	0.0413 (17)	0.0433 (16)	0.0385 (15)	0.0079 (16)	0.0020 (14)	−0.0003 (14)
C14	0.0425 (17)	0.0485 (18)	0.0392 (15)	0.0102 (17)	−0.0026 (14)	−0.0036 (15)
C15	0.052 (2)	0.069 (2)	0.0375 (15)	0.022 (2)	0.0008 (15)	−0.0045 (16)
C16	0.0460 (18)	0.0464 (17)	0.0421 (16)	0.0087 (17)	−0.0037 (15)	−0.0033 (14)
C17	0.0370 (16)	0.0425 (16)	0.0420 (16)	0.0005 (15)	−0.0003 (14)	−0.0008 (14)
C18	0.068 (2)	0.0508 (19)	0.0541 (19)	−0.007 (2)	0.0033 (19)	−0.0030 (17)
C19	0.069 (3)	0.053 (2)	0.063 (2)	−0.005 (2)	−0.002 (2)	0.0078 (18)
C20	0.0406 (17)	0.0491 (18)	0.0408 (16)	0.0034 (16)	0.0011 (14)	−0.0040 (15)
C21	0.0439 (19)	0.089 (3)	0.0490 (18)	0.005 (2)	0.0077 (17)	0.000 (2)
C22	0.0366 (16)	0.0435 (16)	0.0373 (14)	0.0037 (15)	0.0031 (14)	−0.0023 (13)
O22	0.0375 (11)	0.0500 (12)	0.0369 (10)	0.0064 (11)	0.0016 (9)	−0.0021 (10)
C23	0.0383 (17)	0.0507 (18)	0.0399 (16)	0.0009 (15)	0.0032 (15)	−0.0040 (14)
C24	0.0439 (18)	0.061 (2)	0.0485 (17)	0.0003 (18)	−0.0036 (16)	−0.0034 (17)
C25	0.050 (2)	0.059 (2)	0.0530 (19)	0.0090 (19)	−0.0032 (18)	0.0042 (17)
C26	0.061 (2)	0.0443 (18)	0.060 (2)	0.0087 (18)	0.001 (2)	0.0056 (16)
O26	0.0466 (13)	0.0468 (12)	0.0486 (12)	−0.0029 (12)	0.0005 (11)	0.0054 (10)
C27	0.079 (3)	0.079 (3)	0.052 (2)	0.004 (3)	−0.002 (2)	0.015 (2)
O28	0.0521 (13)	0.0620 (14)	0.0480 (12)	−0.0073 (14)	−0.0077 (12)	0.0084 (12)
C29	0.054 (2)	0.059 (2)	0.0481 (18)	0.009 (2)	−0.0064 (17)	−0.0047 (18)
O30	0.0616 (17)	0.0797 (19)	0.0810 (19)	−0.0100 (17)	−0.0189 (16)	0.0064 (17)
C31	0.065 (3)	0.092 (3)	0.056 (2)	0.015 (3)	−0.002 (2)	0.010 (2)
C32	0.0515 (19)	0.0424 (17)	0.0511 (18)	−0.0031 (17)	−0.0078 (17)	−0.0053 (15)
C33	0.064 (2)	0.061 (2)	0.073 (2)	−0.018 (2)	0.000 (2)	0.010 (2)
N34	0.118 (3)	0.0420 (17)	0.096 (3)	−0.013 (2)	−0.039 (3)	0.0033 (18)
O35	0.110 (3)	0.079 (2)	0.123 (3)	−0.018 (2)	0.028 (3)	0.001 (2)

### Geometric parameters (Å, °)

**Table:**

C1—C2	1.519 (5)	C17—C20	1.542 (4)
C1—C10	1.544 (5)	C17—H17C	0.9800
C1—H1A	0.9700	C18—H18C	0.9600
C1—H1B	0.9700	C18—H18D	0.9600
C2—C3	1.520 (5)	C18—H18E	0.9600
C2—H2D	0.9700	C19—H19A	0.9600
C2—H2E	0.9700	C19—H19B	0.9600
C3—O28	1.474 (4)	C19—H19C	0.9600
C3—C4	1.500 (5)	C20—C21	1.520 (5)
C3—H3A	0.9800	C20—C22	1.534 (5)
C4—C5	1.531 (5)	C20—H20A	0.9800
C4—H4A	0.9700	C21—H21B	0.9600
C4—H4B	0.9700	C21—H21C	0.9600
C5—C6	1.529 (5)	C21—H21D	0.9600
C5—C10	1.556 (5)	C22—O26	1.426 (4)
C5—H5A	0.9800	C22—O22	1.427 (4)
C6—C7	1.531 (5)	C22—C23	1.539 (5)
C6—H6A	0.9700	C23—C32	1.509 (5)
C6—H6B	0.9700	C23—C24	1.534 (5)
C7—C8	1.526 (5)	C23—H23C	0.9800
C7—H7B	0.9700	C24—C25	1.522 (5)
C7—H7C	0.9700	C24—H24C	0.9700
C8—C14	1.528 (4)	C24—H24D	0.9700
C8—C9	1.545 (5)	C25—C26	1.509 (5)
C8—H8B	0.9800	C25—C27	1.530 (5)
C9—C11	1.534 (5)	C25—H25C	0.9800
C9—C10	1.565 (4)	C26—O26	1.437 (4)
C9—H9B	0.9800	C26—H26D	0.9700
C10—C19	1.532 (5)	C26—H26E	0.9700
C11—C12	1.537 (5)	C27—H27C	0.9600
C11—H11B	0.9700	C27—H27D	0.9600
C11—H11C	0.9700	C27—H27E	0.9600
C12—C13	1.517 (5)	O28—C29	1.328 (4)
C12—H12A	0.9700	C29—O30	1.210 (5)
C12—H12B	0.9700	C29—C31	1.484 (5)
C13—C18	1.528 (5)	C31—H31B	0.9600
C13—C14	1.549 (4)	C31—H31C	0.9600
C13—C17	1.556 (4)	C31—H31D	0.9600
C14—C15	1.526 (5)	C32—C33	1.477 (5)
C14—H14B	0.9800	C32—N34	1.361 (6)
C15—C16	1.537 (5)	C33—H33A	0.9600
C15—H15B	0.9700	C33—H33B	0.9600
C15—H15C	0.9700	C33—H33C	0.9600
C16—O22	1.437 (4)	N34—H34	0.980 (18)
C16—C17	1.548 (4)	N34—O35	1.367 (5)
C16—H16B	0.9800		
			
C2—C1—C10	116.0 (3)	O22—C16—H16B	110.3
C2—C1—H1A	108.3	C15—C16—H16B	110.3
C10—C1—H1A	108.3	C17—C16—H16B	110.3
C2—C1—H1B	108.3	C20—C17—C16	104.0 (2)
C10—C1—H1B	108.3	C20—C17—C13	121.6 (3)
H1A—C1—H1B	107.4	C16—C17—C13	104.4 (2)
C1—C2—C3	111.4 (3)	C20—C17—H17C	108.7
C1—C2—H2D	109.3	C16—C17—H17C	108.7
C3—C2—H2D	109.3	C13—C17—H17C	108.7
C1—C2—H2E	109.3	C13—C18—H18C	109.5
C3—C2—H2E	109.3	C13—C18—H18D	109.5
H2D—C2—H2E	108.0	H18C—C18—H18D	109.5
O28—C3—C4	105.5 (3)	C13—C18—H18E	109.5
O28—C3—C2	109.0 (3)	H18C—C18—H18E	109.5
C4—C3—C2	111.0 (3)	H18D—C18—H18E	109.5
O28—C3—H3A	110.4	C10—C19—H19A	109.5
C4—C3—H3A	110.4	C10—C19—H19B	109.5
C2—C3—H3A	110.4	H19A—C19—H19B	109.5
C3—C4—C5	113.7 (3)	C10—C19—H19C	109.5
C3—C4—H4A	108.8	H19A—C19—H19C	109.5
C5—C4—H4A	108.8	H19B—C19—H19C	109.5
C3—C4—H4B	108.8	C21—C20—C22	114.9 (3)
C5—C4—H4B	108.8	C21—C20—C17	113.7 (3)
H4A—C4—H4B	107.7	C22—C20—C17	104.2 (3)
C6—C5—C4	110.9 (3)	C21—C20—H20A	107.9
C6—C5—C10	112.0 (3)	C22—C20—H20A	107.9
C4—C5—C10	113.4 (3)	C17—C20—H20A	107.9
C6—C5—H5A	106.7	C20—C21—H21B	109.5
C4—C5—H5A	106.7	C20—C21—H21C	109.5
C10—C5—H5A	106.7	H21B—C21—H21C	109.5
C5—C6—C7	111.6 (3)	C20—C21—H21D	109.5
C5—C6—H6A	109.3	H21B—C21—H21D	109.5
C7—C6—H6A	109.3	H21C—C21—H21D	109.5
C5—C6—H6B	109.3	O26—C22—O22	109.7 (2)
C7—C6—H6B	109.3	O26—C22—C20	106.4 (3)
H6A—C6—H6B	108.0	O22—C22—C20	105.3 (2)
C8—C7—C6	111.7 (3)	O26—C22—C23	110.5 (2)
C8—C7—H7B	109.3	O22—C22—C23	108.6 (2)
C6—C7—H7B	109.3	C20—C22—C23	116.1 (3)
C8—C7—H7C	109.3	C22—O22—C16	106.5 (2)
C6—C7—H7C	109.3	C32—C23—C24	112.1 (3)
H7B—C7—H7C	107.9	C32—C23—C22	112.4 (3)
C7—C8—C14	112.0 (3)	C24—C23—C22	111.1 (3)
C7—C8—C9	111.5 (3)	C32—C23—H23C	107.0
C14—C8—C9	108.4 (3)	C24—C23—H23C	107.0
C7—C8—H8B	108.3	C22—C23—H23C	107.0
C14—C8—H8B	108.3	C25—C24—C23	112.2 (3)
C9—C8—H8B	108.3	C25—C24—H24C	109.2
C11—C9—C8	111.1 (3)	C23—C24—H24C	109.2
C11—C9—C10	114.0 (3)	C25—C24—H24D	109.2
C8—C9—C10	112.2 (3)	C23—C24—H24D	109.2
C11—C9—H9B	106.3	H24C—C24—H24D	107.9
C8—C9—H9B	106.3	C26—C25—C24	107.1 (3)
C10—C9—H9B	106.3	C26—C25—C27	112.7 (3)
C19—C10—C1	106.9 (3)	C24—C25—C27	112.6 (3)
C19—C10—C5	109.4 (3)	C26—C25—H25C	108.1
C1—C10—C5	107.8 (3)	C24—C25—H25C	108.1
C19—C10—C9	110.5 (3)	C27—C25—H25C	108.1
C1—C10—C9	111.8 (3)	O26—C26—C25	111.2 (3)
C5—C10—C9	110.2 (3)	O26—C26—H26D	109.4
C9—C11—C12	113.8 (3)	C25—C26—H26D	109.4
C9—C11—H11B	108.8	O26—C26—H26E	109.4
C12—C11—H11B	108.8	C25—C26—H26E	109.4
C9—C11—H11C	108.8	H26D—C26—H26E	108.0
C12—C11—H11C	108.8	C22—O26—C26	114.2 (3)
H11B—C11—H11C	107.7	C25—C27—H27C	109.5
C13—C12—C11	111.9 (3)	C25—C27—H27D	109.5
C13—C12—H12A	109.2	H27C—C27—H27D	109.5
C11—C12—H12A	109.2	C25—C27—H27E	109.5
C13—C12—H12B	109.2	H27C—C27—H27E	109.5
C11—C12—H12B	109.2	H27D—C27—H27E	109.5
H12A—C12—H12B	107.9	C29—O28—C3	118.9 (3)
C12—C13—C18	110.3 (3)	O30—C29—O28	123.6 (3)
C12—C13—C14	108.0 (3)	O30—C29—C31	125.0 (4)
C18—C13—C14	112.1 (3)	O28—C29—C31	111.3 (4)
C12—C13—C17	114.8 (3)	C29—C31—H31B	109.5
C18—C13—C17	111.7 (3)	C29—C31—H31C	109.5
C14—C13—C17	99.5 (2)	H31B—C31—H31C	109.5
C15—C14—C8	120.3 (3)	C29—C31—H31D	109.5
C15—C14—C13	103.8 (2)	H31B—C31—H31D	109.5
C8—C14—C13	114.1 (3)	H31C—C31—H31D	109.5
C15—C14—H14B	105.8	C23—C32—C33	122.0 (3)
C8—C14—H14B	105.8	N34—C32—C23	111.6 (3)
C13—C14—H14B	105.8	N34—C32—C33	126.3 (3)
C14—C15—C16	101.9 (3)	C32—C33—H33A	109.5
C14—C15—H15B	111.4	C32—C33—H33B	109.5
C16—C15—H15B	111.4	H33A—C33—H33B	109.5
C14—C15—H15C	111.4	C32—C33—H33C	109.5
C16—C15—H15C	111.4	H33A—C33—H33C	109.5
H15B—C15—H15C	109.2	H33B—C33—H33C	109.5
O22—C16—C15	112.6 (3)	C32—N34—O35	109.0 (4)
O22—C16—C17	105.5 (2)	C32—N34—H34	132.0 (16)
C15—C16—C17	107.7 (3)	O35—N34—H34	118.6 (16)
			
C10—C1—C2—C3	54.0 (4)	O22—C16—C17—C20	19.0 (3)
C1—C2—C3—O28	62.8 (4)	C15—C16—C17—C20	139.4 (3)
C1—C2—C3—C4	−53.0 (4)	O22—C16—C17—C13	−109.5 (3)
O28—C3—C4—C5	−63.8 (4)	C15—C16—C17—C13	10.9 (3)
C2—C3—C4—C5	54.2 (4)	C12—C13—C17—C20	93.1 (4)
C3—C4—C5—C6	179.0 (3)	C18—C13—C17—C20	−33.5 (4)
C3—C4—C5—C10	−53.9 (4)	C14—C13—C17—C20	−151.9 (3)
C4—C5—C6—C7	72.1 (4)	C12—C13—C17—C16	−150.0 (3)
C10—C5—C6—C7	−55.8 (5)	C18—C13—C17—C16	83.4 (3)
C5—C6—C7—C8	56.3 (5)	C14—C13—C17—C16	−35.0 (3)
C6—C7—C8—C14	−177.0 (3)	C16—C17—C20—C21	129.4 (3)
C6—C7—C8—C9	−55.3 (4)	C13—C17—C20—C21	−113.5 (3)
C7—C8—C9—C11	−177.2 (3)	C16—C17—C20—C22	3.6 (3)
C14—C8—C9—C11	−53.4 (4)	C13—C17—C20—C22	120.6 (3)
C7—C8—C9—C10	53.9 (4)	C21—C20—C22—O26	−33.8 (4)
C14—C8—C9—C10	177.7 (3)	C17—C20—C22—O26	91.3 (3)
C2—C1—C10—C19	−168.4 (3)	C21—C20—C22—O22	−150.3 (3)
C2—C1—C10—C5	−50.8 (4)	C17—C20—C22—O22	−25.2 (3)
C2—C1—C10—C9	70.5 (4)	C21—C20—C22—C23	89.5 (4)
C6—C5—C10—C19	−68.2 (4)	C17—C20—C22—C23	−145.4 (3)
C4—C5—C10—C19	165.2 (3)	O26—C22—O22—C16	−75.4 (3)
C6—C5—C10—C1	175.9 (3)	C20—C22—O22—C16	38.8 (3)
C4—C5—C10—C1	49.3 (4)	C23—C22—O22—C16	163.8 (3)
C6—C5—C10—C9	53.5 (4)	C15—C16—O22—C22	−153.5 (3)
C4—C5—C10—C9	−73.0 (4)	C17—C16—O22—C22	−36.3 (3)
C11—C9—C10—C19	−58.8 (4)	O26—C22—C23—C32	−176.2 (3)
C8—C9—C10—C19	68.5 (4)	O22—C22—C23—C32	−55.8 (3)
C11—C9—C10—C1	60.1 (4)	C20—C22—C23—C32	62.6 (3)
C8—C9—C10—C1	−172.5 (3)	O26—C22—C23—C24	−49.7 (3)
C11—C9—C10—C5	−179.9 (3)	O22—C22—C23—C24	70.7 (3)
C8—C9—C10—C5	−52.5 (4)	C20—C22—C23—C24	−171.0 (3)
C8—C9—C11—C12	52.7 (4)	C32—C23—C24—C25	177.9 (3)
C10—C9—C11—C12	−179.3 (3)	C22—C23—C24—C25	51.2 (4)
C9—C11—C12—C13	−53.8 (4)	C23—C24—C25—C26	−54.9 (4)
C11—C12—C13—C18	−68.7 (4)	C23—C24—C25—C27	69.6 (4)
C11—C12—C13—C14	54.1 (4)	C24—C25—C26—O26	59.4 (4)
C11—C12—C13—C17	164.1 (3)	C27—C25—C26—O26	−65.0 (4)
C7—C8—C14—C15	−53.3 (4)	O22—C22—O26—C26	−63.2 (3)
C9—C8—C14—C15	−176.8 (3)	C20—C22—O26—C26	−176.7 (3)
C7—C8—C14—C13	−177.7 (3)	C23—C22—O26—C26	56.4 (3)
C9—C8—C14—C13	58.9 (4)	C25—C26—O26—C22	−63.0 (4)
C12—C13—C14—C15	167.9 (3)	C4—C3—O28—C29	−154.6 (3)
C18—C13—C14—C15	−70.4 (3)	C2—C3—O28—C29	86.1 (4)
C17—C13—C14—C15	47.8 (3)	C3—O28—C29—O30	−0.2 (5)
C12—C13—C14—C8	−59.3 (4)	C3—O28—C29—C31	179.7 (3)
C18—C13—C14—C8	62.4 (4)	C24—C23—C32—N34	135.1 (3)
C17—C13—C14—C8	−179.4 (3)	C22—C23—C32—N34	−99.0 (3)
C8—C14—C15—C16	−170.4 (3)	C24—C23—C32—C33	−44.6 (4)
C13—C14—C15—C16	−41.2 (3)	C22—C23—C32—C33	81.3 (4)
C14—C15—C16—O22	134.1 (3)	C33—C32—N34—O35	0.2 (6)
C14—C15—C16—C17	18.3 (4)	C23—C32—N34—O35	−179.5 (3)
